# Quantitative intra-arterial fluorescence angiography for direct monitoring of peripheral revascularization effects

**DOI:** 10.1016/j.jvscit.2025.101770

**Published:** 2025-03-04

**Authors:** Harry G.M. Vaassen, Daan J. Lips, Robert H. Geelkerken, Bryan Wermelink

**Affiliations:** a Fluorescence Imaging Lab, Department of Medical Technology, Medisch Spectrum Twente, Enschede, the Netherlands; b Multimodality Medical Imaging (M3I) group, TechMed Centre, University of Twente, Enschede, the Netherlands; c Department of Surgery, Medisch Spectrum Twente, Enschede, the Netherlands

**Keywords:** Chronic limb-threatening ischemia, Indocyanine green, Quantified fluorescence angiography, Revascularization, Tissue perfusion

## Abstract

**Objective:**

To investigate the feasibility of quantitative fluorescence angiography with intra-arterial dye injection (Q-iaFA) for intraoperative guidance during revascularization procedures in patients with chronic limb-threatening ischemia (CLTI).

**Methods:**

In this observational cohort study, 14 patients with CLTI undergoing endovascular intervention were included. Q-iaFA was performed directly before and after revascularization. The parameters time to peak (TTP) and normalized peak slope (PS_norm_) were derived from intensity-time curves that were measured on the plantar side of the foot in five regions of interest. The main outcome was defined as the change in these Q-iaFA parameters between pre- and postoperative measurements in the region of interest with the most inferior preoperative value. Expected impact of revascularization was classified into strong, moderate or absent, based on intraoperative radiographic imaging and the Trans-Atlantic Inter-Society II standards.

**Results:**

Q-iaFA was successful without complications in all patients. Revascularization impact was classified as strong in 8 (57%), moderate in 5 (36%), and as absent in 1 (7%) patients. In the strong impact group, a significant decrease in TTP and increase in PS_norm_ was observed (*P* = .004). The same trend was less pronounced in the moderate impact group, without statistical significance (*P* = .104 and *P* = .094). Conversely, in the patient with no expected revascularization impact, TTP increased and PS_norm_ decreased.

**Conclusions:**

Q-iaFA is a feasible technique to evaluate peripheral tissue perfusion during vascular interventions. The extracted perfusion parameters are directly affected by revascularization of arterial lesions in patients with CLTI. This finding suggests that Q-iaFA may be useful to guide intraoperative decision making. Work is required to refine quantification strategies and relate Q-iaFA parameters to clinical outcomes.

Chronic limb-threatening ischemia (CLTI) is a critical condition characterized by reduced blood flow, below the minimal metabolic demand, to the lower limbs. The condition is reflected clinically as rest pain, nonhealing ulcerations, and gangrene. CLTI is associated with strongly heightened cardiovascular mortality and its increasing prevalence is an urgent issue that imposes a substantial strain on health care worldwide.^1^^,^^2^ Treatment usually requires revascularization procedures to achieve limb salvage.^3^ Despite best efforts, amputation rates are still reported at 15% to 20% at 1 year after diagnosis.^1^

A currently missing piece in revascularization procedures is the ability to monitor how treatment affects tissue perfusion in real time.^4^^,^^5^ In the operating room, focus lies on macrovascular imaging and there is limited insight on whether microvascular perfusion is actually being improved.^6^ The interventionist may therefore be too aggressive, for example, by taking unnecessary risks attempting to treat crural arteries or, conversely, exercising excessive caution, leading to undertreatment.^4^ A technological aid is required to assist in these decisions.

Unfortunately, at present no technique can be recommended to serve this purpose.^5^ The accuracy and intraoperative applicability of the ankle-brachial index and toe pressure are limited,^7^^,^^8^ and newer methods such as laser speckle imaging, hyperspectral imaging and two-dimensional perfusion angiography are still lacking in imaging depth, reproducibility, and predictive power for clinical outcome.^9^^,^^10^

Fluorescence angiography (FA) is a widely upcoming method for evaluating vascularity and tissue perfusion. It comprises the administration of the fluorescent contrast agent indocyanine green (ICG) into the blood stream, illumination of the tissue under investigation with near-infrared (NIR) light, and detection of the emitted fluorescence using a camera. The ability of NIR light to penetrate tissue, combined with the real-time imaging of its fluorescent interaction with ICG, allows observers to monitor the movement of the contrast agent through organ vasculature, providing insights about tissue perfusion. The approach is being implemented successfully in fields such as gastrointestinal surgery and reconstructive surgery.^11^^,^^12^ Also, vascular surgery studies have shown the potential of the technique as a diagnostic modality or intraoperative tool.^13^, ^14^, ^15^

The use of quantitative parameters (Q-FA) is encouraged when analyzing FA images to minimize significant interobserver variability.^16^ In particular, dynamic time-based parameters should be more reliable than parameters based on fluorescence intensity, because they are less affected by external factors such as camera-to-object distance and ambient light.^17^ In almost all instances, ICG is injected in a peripheral vein as a bolus, in accordance with the US Food and Drug Administration-prescribed administration route.^18^ However, while the bolus travels from the injection site through the cardiopulmonary system to the target organ, unwanted variation in the bolus shape and subsequent Q-FA parameters can arise.^19^ In addition, a relatively high amount of contrast agent is required, which remains in circulation and hampers repeated measurements. Direct ICG injection in the feeding artery can circumvent these issues potentially, but is unfeasible for most target organs owing to the lack of access. Fortunately, surgical and endovascular intervention for CLTI provides access to the common femoral artery (CFA) without additional invasiveness. A study by Igari et al^20^ previously demonstrated Q-FA with intra-arterial ICG injection (Q-iaFA) in patients with peripheral arterial disease, where it was observed that the extracted parameters correlate with traditional perfusion measures such as the ankle-brachial index.

The purpose of this study was to investigate the feasibility of Q-iaFA for intraoperative use during CLTI treatment, by relating the technical success of the intervention to Q-iaFA perfusion parameters.

## Methods

### Study design

This observational pilot study was performed at a single centre specialized in endovascular treatment of CLTI. In total, fifteen patients were included between February and June 2024. Patients were eligible for inclusion when they were newly diagnosed with CLTI (Rutherford classification ≥4) and scheduled for endovascular intervention. Patients were not included if the surgical plan involved a common femoral endarterectomy, because this would hinder a representative baseline measurement. Other exclusion criteria were age less than 18 years, known allergy or hypersensitivity for ICG and/or iodine, hyperthyroidism, and severely impaired renal function (estimated glomerular filtration rate of <30 mL/min). The study adhered to the ethics guidelines of the Declaration of Helsinki and ethical approval was granted by the Medical research Ethics Committee United (Nieuwegein, the Netherlands) under reference R22.100. All patients provided written informed consent. To enforce the observational nature of this study, the surgical team was blinded for all Q-iaFA images.

### Data collection

**Fig 1:**
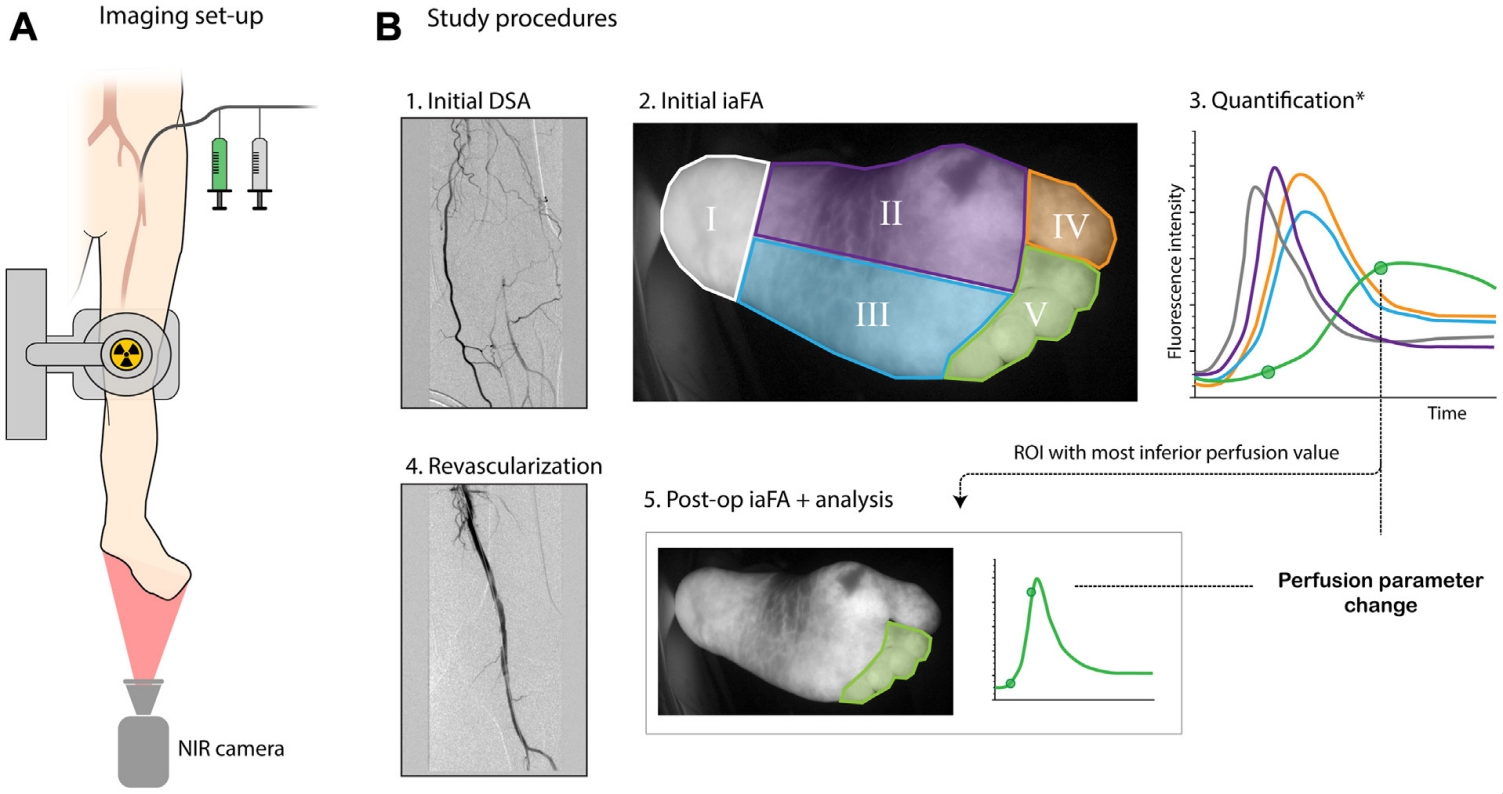
Study methodology. **(A)** Intraoperative imaging setup, including common femoral artery puncture access for administration of indocyanine green and x-ray contrast agent. **(B)** Intraoperative study procedures, where Q-iaFA is performed after initial DSA and after the revascularization procedure is ended. Perfusion parameters were quantified in five ROIs: the main outcome was the change in perfusion parameter in the ROI with the most inferior preoperative value.∗Time-intensity curves are illustrative and not extracted from experimental data. *DSA*, Digital subtraction angiogram; *NIR*, near-infrared; *Q-iaFA*, (quantified) fluorescence angiography with intra-arterial injection of dye; *ROI*, region of interest.

All procedures were performed under general anaesthesia. On each patient, Q-iaFA was performed twice: once after introduction of a 6 Fr endovascular sheath (Cordis, Hialeah, FL) in the CFA and once after the operating surgeon had decided to end the procedure. ICG (Verdye, Diagnostic Green Ltd, Westmeath, Ireland) was administered in a dosage of 0.01 mg/kg as a 1-mL bolus through the CFA sheath, which was immediately flushed with 10 mL NaCl thereafter. NIR imaging was performed using the Spectrum 3 system (Quest Medical Imaging, Middenmeer, the Netherlands), using an exposure time and gain of 100 ms and 21 dB, respectively. The system recorded at 4 frames per second for a duration of 3 minutes after ICG injection. The camera head was aimed at the plantar side of the foot at a distance of 30 cm (Fig 1, *A*). Ambient light in the operating room was minimized.

Basic patient characteristics and cardiovascular risk factors were extracted from medical records. Global Limb Anatomic Staging System (GLASS) scores^21^ were determined based on the initial intraoperative digital subtraction angiogram (DSA) and—if required—on a preoperative computed tomography angiography scan. The expected technical impact of the revascularization on tissue perfusion was classified into one of three categories based on intraoperative DSA imaging and the Trans-Atlantic Inter-Society Consensus (TASC) II classification standard.^22^ A strong impact required successful revascularization of an aortoiliac or femoropopliteal TASC II type C or D lesion and the presence of at least one clear crural artery pathway to the pedal arc. The revascularizations of a type A or B lesion, or cases without a clear pathway to the pedal arc, were classified as moderate impact. Expected impact in procedures without significant revascularization (residual stenosis >30%) was classified as absent.^23^

### Fluorescence imaging analysis

Analysis of Q-iaFA imaging data and calculation of perfusion parameters was performed using in-house built software.^24^ Five regions of interest (ROIs) were drawn: the posterior (I) and the medial (II) and lateral (III) parts of the foot, as well as the hallux (IV) and digits 2 to 5 (V) (Fig 1, *B*). Time-intensity curves were extracted by taking the mean intensity in those regions for each video frame.

Two perfusion parameters were defined. Time to peak (TTP) was calculated as the time between the points where the curve reached 10% and 90% of maximum intensity, to avoid ambiguity in finding the exact start and end point of the intensity rise. The peak slope (PS) of the signal was measured after applying a moving average filter with a window size of 2 seconds to omit noise. This value of PS was subsequently normalized by dividing with the total increase in intensity, to obtain normalized PS (PS_norm_). It was expected that revascularization and the presumed increase in tissue perfusion would result in a decrease in TTP and an increase in PS_norm_.

The main end point of this study was the change in Q-iaFA perfusion parameters before and after revascularization. For each patient, the ROI that had the most inferior perfusion value (ie highest TTP, lowest PS_norm_) in the preoperative video was selected and used for this analysis.

The statistical library of SciPy was used for statistical analysis.^25^ Descriptive statistics of continuous variables are presented as median and interquartile range (IQR). Differences in paired data were tested for significance using the one-sided Wilcoxon signed-rank test.

## Results

All study procedures were completed in 14 patients. One patient was excluded because the revascularization procedure had to be terminated prematurely owing to ongoing cardiopulmonary instability. There were no adverse events related to arterial ICG injection. The baseline demographics and procedure details are depicted in Table I. Overall, the iliac and crural artery tracts were each targeted in two procedures (14%), the SFA in nine procedures (64%), and the popliteal artery in seven procedures (50%).

**Table I:** Patient demography and procedure details

	Strong (n = 8)	Moderate (n = 5)	Absent (n = 1)	Total (n = 14)
Age, years	76 ± 10	77 ± 13	69	76 ± 10
Male gender	2 (25)	3 (60)	1 (100)	6 (43)
BMI kg/m^2^	31 ± 16	25 ± 5	32	29 ± 13
ASA score				
II	0 (0)	2 (40)		2 (14)
III	5 (63)	2 (40)		7 (50)
IV	3 (27)	1 (20)	1 (100)	5 (36)
Diabetes	3 (38)	2 (40)	1 (100)	6 (43)
Hypertension	7 (88)	4 (80)	1 (100)	12 (86)
Coronary disease	2 (25)	0 (0)	1 (100)	3 (21)
Smoking history	7 (88)	3 (60)	1 (100)	11 (79)
Revascularization target				
Iliac	1 (13)	1 (20)	0 (0)	2 (14)
SFA	7 (88)	2 (40)	0 (0)	9 (64)
Popliteal	4 (50)	3 (60)	0 (0)	7 (50)
Crural	0 (0)	1 (20)	1 (0)	2 (14)
Rutherford stage				
IV	2 (33)	1 (20)	0 (0)	3 (21)
V	6 (67)	4 (80)	1 (100)	11 (79)
GLASS stage				
II	5 (63)	4 (80)	0 (0)	9 (64)
III	3 (37)	1 (20)	1 (100)	5 (36)

*ASA,* American Society of Anesthesiologists; *BMI,* body mass index; *GLASS,* Global Limb Anatomic Staging System; *SFA,* superior femoral artery.

Values are mean ± standard deviation or number (%).

**Fig 2:**
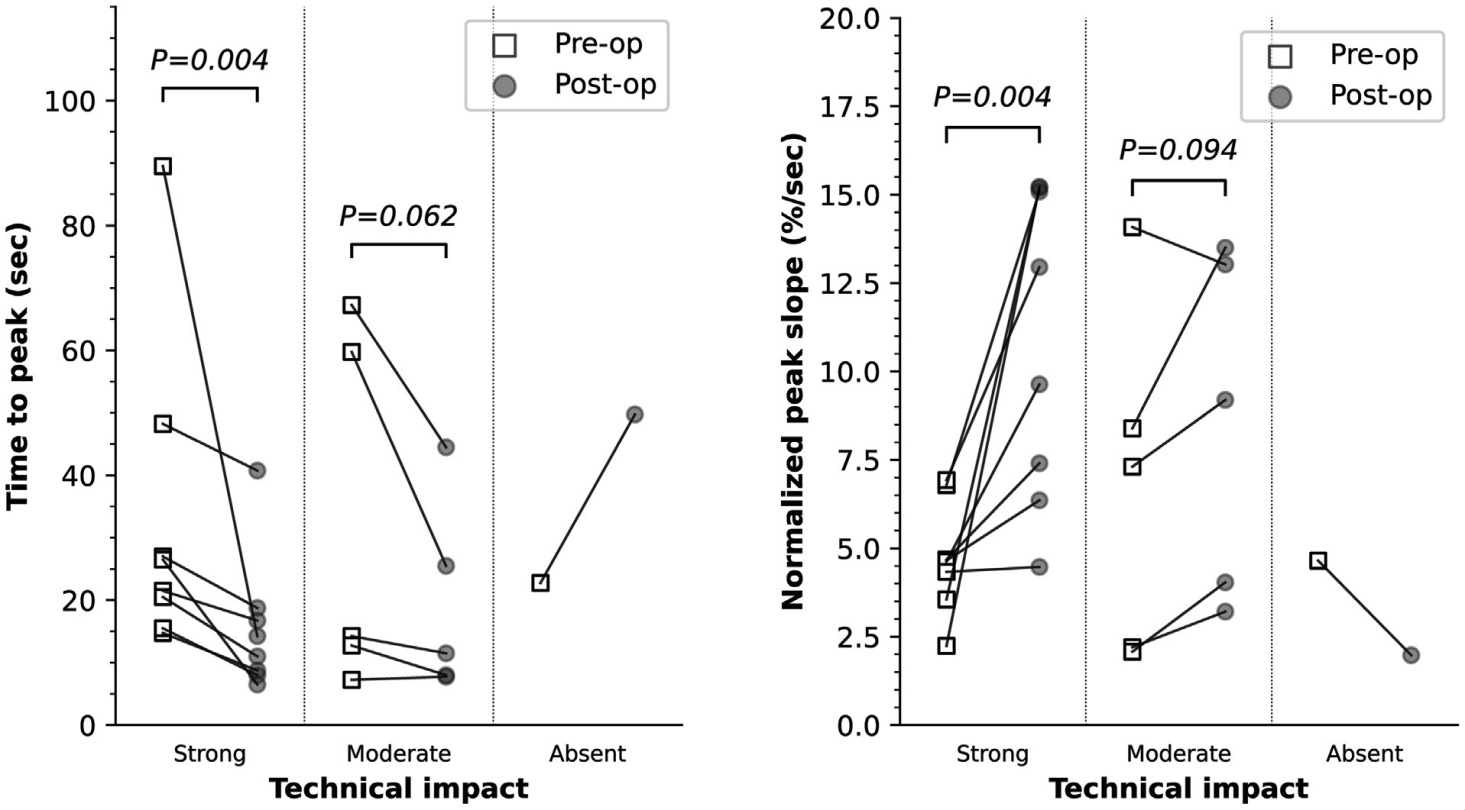
Scatter plots depicting the quantification of time to peak (TTP) and normalized peak slope (PS) for pairwise preoperative and postoperative measurements. Measurements were extracted from the region of interest (ROI) with the most inferior preoperative perfusion parameter value. *P* values were determined with a one-sided Wilcoxon signed-rank test.

Revascularization impact was classified as strong in eight (57%), moderate in five (36%), and as absent in one (7%) patients. ROI II contained the most inferior preoperative perfusion value in one (7%) patient, ROI III in three patients (21%), ROI IV in five patients (36%), and ROI V in five patients (36%). Table II presents the overall Q-iaFA quantification results. The pairwise preoperative and postoperative measurements in the most inferior ROI are depicted in Fig 2. The median TTP significantly decreased from 24.0 seconds (IQR, 13.1 seconds) to 12.6 seconds (IQR, 8.7 seconds) (*P* = .004) and PS_norm_ significantly increased from 4.6%/second (IQR, 1.1%/second) to 11.3%/second (IQR, 7.8%/second) (*P* = .004) in the strong impact group. In the moderate impact group, the median TTP decreased from 14.3 seconds (IQR, 47.0 seconds) to 11.5 seconds (IQR, 17.5 seconds) and PS_norm_ increased from 7.3%/second (IQR, 6.1%/second) to 9.2 %/second (IQR, 9.0%/second), both without reaching statistical significance. Notably, the patient with absent suspected impact showed an increase in TTP from 22.8 to 49.8 seconds and a decrease in PS_norm_ from 4.7%/second to 2.0%/second.

**Table II:** Analysis of quantitative fluorescence angiography with intra-arterial dye injection (Q-iaFA) data

	Strong (n = 8)	Moderate (n = 5)	Absent (n = 1)
TTP, s (all ROIs)			
Before revascularization	15.3 (11.6)	10.8 (23.0)	17.3 (6.8)
After revascularization	9.4 (8.8)	10.5 (17.5)	39.8 (24.8)
*P* value	<.001	.104	
Relative change, %	−26.7 (45.9)	0.0 (38.2)	18.7 (16.5)
TTP, s (inferior ROI)			
Before revascularization	24.0 (13.1)	14.3 (47.0)	22.8
After revascularization	12.6 (8.7)	11.5 (17.5)	49.8
*P* value	.004	.062	
Relative change, %	−43.5 (26.7)	−33.8 (18.0)	18.7
PS_norm_ (all ROIs), %/s			
Before revascularization	8.3 (3.2)	10.9 (10.9)	6.6 (3.1)
After revascularization	11.4 (7.9)	9.6 (9.2)	2.9 (2.7)
*P* value	<.001	.479	
Relative change, %	35.8 (99.6)	2.3 (36.4)	−55.4 (8.0)
PS_norm_ (inferior ROI), %/s			
Before revascularization	4.6 (1.1)	7.3 (6.1)	4.7
After revascularization	11.3 (7.8)	9.2 (9.0)	2.0
P value	0.004	0.094	
Relative change, %	97.7 (120.8)	45.8 (35.0)	−57.4

*PS*_*norm*_*,* Normalized peak slope; *ROI,* region of interest; *TTP,* time to peak.

Values are median (interquartile range).

Considering the preoperative measurement in all ROIs, patients with diabetes exhibited a significantly higher median TTP (22.1 seconds vs 13.0 seconds; *P* = .013) and lower median PS_norm_ (7.0%/second vs 12.3%/second; *P* = .001) than patients without diabetes. There was no significant difference in Q-iaFA parameter improvement when only the ROI with the most inferior preoperative value was examined.

## Discussion

CLTI is a pressing health issue that requires interventions to promote amputation-free survival. New methods are needed to guide clinical decision-making and improve patient outcomes. FA with ICG is a safe and accessible method to assess tissue perfusion that is gaining traction in various surgical disciplines. This study illustrates the feasibility of Q-iaFA as a method to promptly monitor the effects of revascularization procedures on local tissue perfusion in the operating room.

Previous work on intravenous Q-FA in patients with CLTI demonstrated improvement in perfusion parameters after revascularization, but also revealed a large variation between patients, which complicates threshold values.^13^^,^^14^ Likewise, although Tange et al^26^ found a clear correlation between improvement of Q-FA parameters and clinical outcome, predictive accuracy remained limited (area under receiver operating curve <0.8).

Igari et al^20^ showed the concept of Q-iaFA in peripheral arterial disease and found a significant correlation between Q-iaFA parameters and traditional measurements such as the ankle-brachial index and ankle pressure. The present study extrapolates this concept to direct intraoperative evaluation of revascularization effects in patients with CLTI. We found that Q-iaFA perfusion parameters are immediately affected by revascularization of substantial arterial flow-restricting lesions. The findings indicate that these parameters are indeed a reflection of local tissue perfusion in the foot, and that they might be suitable to substantiate intraoperative decisions. This reflection is supported by the observation that revascularizations with strong suspected impact showed more pronounced improvement in Q-iaFA perfusion parameters than those with moderate or no suspected impact. It should, however, be noted that the group sizes in this pilot study are too small for comprehensive subanalyses.

Despite the approved administration route of ICG being intravenous, intra-arterial injection makes intuitive sense when the feeding artery is accessible. Considering the facts that the used dosage of 0.01 mg/kg is extremely low compared with the maximum daily dosage of ICG (5 mg/kg), and that ICG does not interact with tissues, there is no reason to suspect that an intra-arterial injection would raise safety concerns. The advantages of intra-arterial dye administration are evident, because the necessary information on inflow and outflow could be extracted within 3 minutes after injection. Furthermore, the low total dose resulting in faster systemic clearance allows quickly repeated measurements. This factor makes it possible for interventionists to use Q-iaFA along with traditional DSA. In this setup, DSA could provide information on the technical possibilities of revascularization, and Q-iaFA may indicate its clinical necessity for, for example, wound healing. In the case of multilevel lesions, the interventionist can perform these diagnostics repeatedly and iteratively evaluate whether additional revascularization is appropriate. Future prospective studies should, therefore, relate Q-iaFA parameters to clinical outcomes, possibly establishing a baseline where further intervention is not indicated.

Intra-arterial administration also increases the repeatability of the ICG bolus shape that arrives at the foot. Nevertheless, even patients classified in the strong impact group—and thus having a clear inflow into the pedal arc—showed postoperative perfusion values that overlap with preoperative values from other patients. The quantification methodology, therefore, still requires attention. Improvement might be pursued in various ways. First, the relative change in preoperative and postoperative perfusion parameters may be a more robust metric than absolute values. Relative measures are less susceptible to interpatient variation, but may be difficult to relate to clinical outcome. Future studies should identify parameters with the greatest clinical predictive value. Second, a healthy range extracted from patients without vascular disease is desirable. Finally, the selection of ROIs can be further investigated. In this study, we opted for the plantar side of the foot, because it is logistically convenient in the operating room, and defined five ROIs based on the angiosome concept. However, other strategies are available, such as focusing specifically on wound areas in Rutherford class V and VI patients.

Several limitations apply to this study, most prominently the limited number of patients. This sample size was sufficient to demonstrate the feasibility and potential of Q-iaFA, but larger studies are required to dive deeper into quantification strategies, threshold values, and ultimately clinical outcomes. We furthermore estimated the suspected impact of a revascularization procedure using a classification scheme that is novel and thus not externally validated. It is, however, based on the recognized TASC II and GLASS definitions.^3^^,^^22^ To our knowledge, there is currently no suitable scoring system to anatomically grade revascularization procedures on their suspected impact on local perfusion.

To conclude, Q-iaFA with ICG is an easy-to-use method that can swiftly provide useful information on the perfusion state of the foot in an intraoperative setting. Arterial injection delivers relevant advantages over intravenous injection and is, therefore, advised when using Q-FA during peripheral revascularization procedures. Further work is necessary to optimize quantification strategies related to patient-reported outcome measurements.

## Funding

Quest Medical Imaging facilitated the use of a fluorescence imaging device for the purpose of this study free of charge. This party had no involvement the study design, manuscript, decision to submit and/or any other relevant aspects.

## Disclosures

None.

## References

[1] Duff S., Mafilios M.S., Bhounsule P., Hasegawa J.T. (2019). The burden of critical limb ischemia: a review of recent literature. Vasc Health Risk Manag.

[2] Barnes J.A., Eid M.A., Creager M.A., Goodney P.P. (2020). Epidemiology and risk of amputation in patients with diabetes mellitus and peripheral artery disease. Arterioscler Thromb Vasc Biol.

[3] Conte M.S., Bradbury A.W., Kolh P. (2019). Global vascular guidelines on the management of chronic limb-threatening ischemia. Eur J Vasc Endovascular Surg.

[4] Brouwers J., van Rijswijk C., Van Den Hoven P., Hamming J., van der Vorst J.R. (2023). Chronic limb-threatening ischemia: when is enough enough?. J Endovasc Ther.

[5] Wermelink B., Ma K.F., Haalboom M., El Moumni M., de Vries J.P.P.M., Geelkerken R.H. (2021). A systematic review and critical appraisal of peri-procedural tissue perfusion techniques and their clinical value in patients with peripheral arterial disease. Eur J Vasc Endovasc Surg.

[6] Li W.W., Carter M.J., Mashiach E., Guthrie S.D. (2017). Vascular assessment of wound healing: a clinical review. Int Wound J.

[7] de Graaff J.C., Ubbink DTh, Legemate D.A., de Haan R.J., Jacobs M.J.H.M. (2001). Interobserver and intraobserver reproducibility of peripheral blood and oxygen pressure measurements in the assessment of lower extremity arterial disease. J Vasc Surg.

[8] Wyman R.A., Keevil J.G., Busse K.L., Aeschlimann S.E., Korcarz C.E., Stein J.H. (2006). Is the ankle-brachial index a useful screening test for subclinical atherosclerosis in asymptomatic, middle-aged adults?. WMJ.

[9] Kleiss S.F., Ma K.F., El Moumni M. (2023). Detecting changes in tissue perfusion with hyperspectral imaging and thermal imaging following endovascular treatment for peripheral arterial disease. J Endovasc Ther.

[10] Wermelink B., Mennes O.A., Van Baal J.G. (2022). Assessing the microcirculation of the foot with laser speckle contrast imaging during endovascular and hybrid revascularisation procedures in patients with chronic limb threatening Ischaemia. Eur J Vasc Endovasc Surg.

[11] Wexner S., Abu-Gazala M., Boni L. (2022). Use of fluorescence imaging and indocyanine green during colorectal surgery: results of an intercontinental Delphi survey. Surgery.

[12] Schols R.M., Dip F., Lo Menzo E. (2022). Delphi survey of intercontinental experts to identify areas of consensus on the use of indocyanine green angiography for tissue perfusion assessment during plastic and reconstructive surgery. Surgery.

[13] Settembre N., Kauhanen P., Albäck A., Spillerova K., Venermo M. (2017). Quality control of the foot revascularization using indocyanine green fluorescence imaging. World J Surg.

[14] Van den Hoven P., S Weller F., Van De Bent M. (2022). Near-infrared fluorescence imaging with indocyanine green for quantification of changes in tissue perfusion following revascularization. Vascular.

[15] van den Hoven P., Ooms S., van Manen L. (2019). A systematic review of the use of near-infrared fluorescence imaging in patients with peripheral artery disease. J Vasc Surg.

[16] Hardy N.P., Dalli J., Khan M.F., Andrejevic P., Neary P.M., Cahill R.A. (2021). Inter-user variation in the interpretation of near infrared perfusion imaging using indocyanine green in colorectal surgery. Surg Endosc.

[17] Lütken C.D., Achiam M.P., Svendsen M.B., Boni L., Nerup N. (2020). Optimizing quantitative fluorescence angiography for visceral perfusion assessment. Surg Endosc.

[18] Alander J.T., Kaartinen I., Laakso A. (2012). A review of indocyanine green fluorescent imaging in surgery. Int J Biomed Imaging.

[19] Elliott J.T., Addante R.R., Slobegean G.-P. (2020). Intraoperative fluorescence perfusion assessment should be corrected by a measured subject-specific arterial input function. J Biomed Opt.

[20] Igari K., Kudo T., Uchiyama H., Toyofuku T., Inoue Y. (2014). Intraarterial injection of indocyanine green for evaluation of peripheral blood circulation in patients with peripheral arterial disease. Ann Vasc Surg.

[21] Wijnand J.G.J., Zarkowsky D., Wu B. (2021). The global limb anatomic staging system (GLASS) for CLTI: improving inter-observer agreement. J Clin Med.

[22] Norgren L., Hiatt W.R., Dormandy J.A., Nehler M.R., Harris K.A., Fowkes F.G.R. (2007). Inter-society consensus for the management of peripheral arterial disease (TASC II). J Vasc Surg.

[23] Diehm N., Pattynama P.M., Jaff M.R. (2008). Clinical endpoints in peripheral endovascular revascularization trials: a case for standardized definitions. Eur J Vasc Endovascular Surg.

[24] Vaassen H.G.M., Wermelink B., Manohar S., Geelkerken R.H., Lips D.J. (2022). Intraoperative quantification of fluorescence angiography for assessment of intestinal perfusion: *in vivo* exploration of clinical value. BJS Open.

[25] Virtanen P., Gommers R., Oliphant T.E. (2020). SciPy 1.0: fundamental algorithms for scientific computing in python. Nat Methods.

[26] Tange F.P., van den Hoven P., van Schaik J. (2024). Near-infrared fluorescence imaging with indocyanine green to Predict clinical outcome after revascularization in lower extremity arterial disease. Angiology.

